# The cytosine methylation landscape of spring barley revealed by a new reduced representation bisulfite sequencing pipeline, WellMeth

**DOI:** 10.1002/tpg2.20049

**Published:** 2020-09-14

**Authors:** Marta Malinowska, Istvan Nagy, Cornelis A. M. Wagemaker, Anja K. Ruud, Simon F. Svane, Kristian Thorup‐Kristensen, Christian S. Jensen, Birger Eriksen, Lene Krusell, Ahmed Jahoor, Jens Jensen, Lars Bonde Eriksen, Torben Asp

**Affiliations:** ^1^ Department of Molecular Biology and Genetics Aarhus University Slagelse Denmark; ^2^ Center for Quantitative Genetics & Genomics Aarhus University Slagelse Denmark; ^3^ Department of Experimental Plant Ecology Radboud University Nijmegen The Netherlands; ^4^ Department of Plant and Environmental Science University of Copenhagen Frederiksberg Denmark; ^5^ Sejet Plant Breeding Horsens Denmark; ^6^ Nordic Seed A/S Galten Denmark; ^7^ Research Division DLF Seeds A/S Store Heddinge Denmark; ^8^ Landbrug & Fødevarer SEGES Aarhus Denmark; ^9^ LIMAGRAIN A/S Horsens Denmark

## Abstract

Patterns and level of cytosine methylation vary widely among plant species and are associated with genome size as well as the proportion of transposons and other repetitive elements in the genome. We explored epigenetic patterns and diversity in a representative proportion of the spring barley (*Hordeum vulgare* L.) genome across several commercial and historical cultivars. This study adapted a genotyping‐by‐sequencing (GBS) approach for the detection of methylated cytosines in genomic DNA. To analyze the data, we developed WellMeth, a complete pipeline for analysis of reduced representation bisulfite sequencing. WellMeth enabled quantification of context‐specific DNA methylation at the single‐base resolution as well as identification of differentially methylated sites (DMCs) and regions (DMRs). On average, DNA methylation levels were significantly higher than what is commonly observed in many plants species, reaching over 10‐fold higher levels than those in *Arabidopsis thaliana* (L.) Heynh. in the CHH methylation. Preferential methylation was observed within and at the edges of long‐terminal repeats (LTR) retrotransposons *Gypsy* and *Copia*. From a pairwise comparison of cultivars, numerous DMRs could be identified of which more than 5,000 were conserved within the analyzed set of barley cultivars. The subset of regions overlapping with genes showed enrichment in gene ontology (GO) categories associated with chromatin and cellular structure and organization. A significant correlation between genetic and epigenetic distances suggests that a considerable portion of methylated regions is under strict genetic control in barley. The data presented herein represents the first step in efforts toward a better understanding of genome‐level structural and functional aspects of methylation in barley.

**Abbreviations**
DMC: differentially methylated site
DMR: differentially methylated region
FPKM: fragments per kilobase million
GBS: genotyping‐by‐sequencing
GO: gene ontology
LTR: long‐terminal repeat
PC: principal component
PCA: principal component analysis
PCR: polymerase chain reaction
RRBS: reduced representation bisulfite sequencing
TE: transposable element
TES: transcription end sites
TSS: transcription start sites
UMI: unique molecular identifier
UTR: untranslated region
WGBS: whole‐genome bisulfite sequencing.

## 1 INTRODUCTION

Barley is one of the four major cereals grown over about 47 million ha worldwide and with a world production over 147 Tg in 2017 (FAOSTAT, 2018). It is used for human and animal consumption and for producing malt for the distilling and brewing industry. Barley is cultivated across a wide range of environments, which can be attributed to its adaptability to different climates and high tolerance to various abiotic stresses (Newton et al., 2011). Anthropogenic climate change is a threat to food security, and through direct (water and nutrient availability and abiotic stresses) and indirect (diseases and pests) impacts is expected to cause further barley yield stagnation (Ray et al., 2019; Schauberger et al., 2018). In the face of changing climate and a growing human population, quality and yield improvement under adverse environmental conditions are important goals in barley breeding programs in northern Europe. Since genetic diversity is limited, it is of high interest to explore the prospect of including epigenetics to assist barley breeding strategies.

DNA methylation is a dynamic epigenetic modification playing a role in the regulation of the plant genome during environmental adaptation and development and is associated with cellular processes like the regulation of transposon silencing or expression (Law & Jacobsen, 2010; Miura et al., 2001; Pikaard & Scheid, 2014; Zhang, Lang, & Zhu, 2018). Patterns in DNA methylation can manifest as a result of genetic control, environmental stimuli, or stochastic epigenetic alteration. Most of the changes to patterns of DNA methylation are reversible or of no consequence but some could be passed down through meiosis and persist between generations (Heard & Martienssen, 2014). The reversible nature of DNA methylation is thought to be a source of phenotypic plasticity, which, under very dynamic environments, may benefit the survival and reproduction of populations (Springer, 2013). Heritable epigenetic variants that affect phenotypes could be under natural selection and contribute to adaptation independently of genetic variation (Richards et al., 2017).

In plants, DNA methylation occurs in both symmetric CG and CHG, and asymmetric CHH (where H is A, T or C) contexts (Meyer, Niedenhof, & Ten Lohuis, 1994). The methylation state reflects a balance between distinct molecular pathways that establish de novo, maintain, or remove methylation and, in the cases of CHG and CHH, are unique to plants (Law & Jacobsen, 2010; Stroud et al., 2014). De novo establishment of DNA methylation is mediated by the RNA‐directed DNA methylation pathway (Law & Jacobsen, 2010). After DNA replication, maintenance of DNA methylation, depending on the cytosine sequence context, is regulated by multiple methyltransferases (Law & Jacobsen, 2010). The role and effect of methylation depend on the pattern, context, and complexity of the genome as well as chromatin configuration (Law & Jacobsen, 2010). Studies have shown that DNA methylation plays an important role in (a) the regulation of plant developmental processes and (b) the response to biotic and abiotic environmental stimuli (Cubas et al., 1999; Le et al., 2014; Manning et al., 2006).

Despite a growing interest in the impact of epigenetic variation on crop performance under different environmental conditions (Gallusci et al., 2017; Richards et al., 2017; Springer, 2013), there have been very few studies focusing on DNA methylation landscape diversity in barley. Studies of epigenetic variation published in the last 10 yr have used methylation‐sensitive amplification polymorphism (Chwialkowska, Nowakowska, Mroziewicz, Szarejko, & Kwasniewski, 2016) for the detection of methylome modulation under drought, methylation‐sensitive restriction enzyme, multiplexed polymerase chain reaction (PCR) for responses to salt stress, and bisulfite sequencing for responses to salt (Konate et al., 2018) or heavy metals (Kashino‐fujii et al., 2018).

Multiple methods have been developed to investigate DNA methylation patterns, levels, or variation on a genome‐wide scale Kurdyukov & Bullock, (2016). Since the first demonstration of using bisulfite conversion of 5‐methylcytosine in sequencing Frommer et al., (1992), the application of high‐throughput, next‐generation bisulfite sequencing technologies has accelerated the development of powerful epigenetic tools. With the advantage of targeting essentially every cytosine base in a genome, the whole‐genome bisulfite sequencing (WGBS) (Cokus et al., 2008; Lister et al., 2010) is considered the gold standard for a comprehensive analysis of DNA methylation variation. However, aside from being restricted to species where there is a high‐quality genome available, it is also the most expensive method to study genome‐wide methylation. A cost‐effective alternative to WGBS is reduced representation bisulfite sequencing (RRBS) that uses a fraction of the genome and generates a scalable DNA methylation profile at single‐nucleotide resolution (Gu et al., 2011).

By using double‐enzyme digestion in the RRBS method, it is possible to increase coverage of the detected regions more accurately and thus reflect their average methylation levels (Wang et al., 2013). In recent years, updated reduced representation approaches such as bsRADseq (Trucchi, Mazzarella, Gilfillan, Lorenzo, & O, 2016) and epiGBS (Van Gurp et al., 2016) have been developed for species lacking a reference genome. While these methods may be attractive for particular population and metagenomic studies, the tedious steps of constructing synthetic references (reconstructing the original sequences from stranded bisulfite‐treated reads), and subsequent difficulties of resolving their genomic positions, are rather objectionable for species with complex genomes and available genomic resources.

Here we present WellMeth, a complete RRBS analysis pipeline for species with high‐quality reference genomes. In the study, we examine and compare the methylation landscape of 12 commercial and historical spring barley cultivars and lay the groundwork to better understand variation in the barley genome beyond differences in DNA sequence.

**Core Ideas**

- Presents WellMeth, a complete, reduced representation bisulfite sequencing analysis pipeline.
- Assess context‐specific DNA methylation patterns in 12 cultivars.
- Spring barley has a very high proportion of CHH methylation throughout the genome.
- Preferential methylation of transposable elements was observed.
- Variation in methylation profiles partially mirrored the genetic diversity of the cultivars.

## 2 MATERIALS AND METHODS

### 2.1 Plant material

Twelve spring barley commercial cultivars (Evelina, Invictus, Kenia, Prisma, Tocada, Evergreen, Laurikka, Flair, Laureate, RGTPlanet, Kaarle and Alvari) were grown in the RadiMax facility located at Copenhagen University's experimental farm, west of Copenhagen, Denmark (Svane, Jensen, & Kristensen, 2019). Flag leaves from seven different plants per cultivar, were collected before heading. Extraction and further analysis of DNA and RNA was conducted for one pool of seven individuals per cultivar (*n* = 12).

### 2.2 Library preparation

Genomic DNA was extracted by using the standard cetyltrimethyl‐ammonium‐bromide (CTAB) method, quantified and checked for quality using the PicoGreen dsDNA Assay Kit. EpiGBS libraries were prepared as described by Van Gurp et al. (2016). Briefly, genomic DNA (400 ng) was digested using two methylation insensitive restriction endonucleases PacI (New England BioLabs, Inc.) and Nsil (New England BioLabs, Inc.). After digestion, barcoded adaptors were ligated to the fragments using T4 DNA ligase (New England BioLabs, Inc.). An equal volume of 12 ligates were pooled and purified using PCR cleanup (Marchery Nagel) followed by size selection using AMPureXP (Beckman Coulter, Inc.) according to the manufacturer's protocol and favoring DNA fragments >200 bp,. Prior to bisulfite treatment, we carried out nick translation with DNA polymerase I (New England BioLabs, Inc.) and 5‐methylcytosine dNTP mix (Zymo Research). A nick, in each fragment–adapter connection, was created because the barcoded adapters were not phosphorylated to prevent the formation of adapter dimers. The nick‐translated libraries were treated with bisulfite to convert unmethylated (but not methylated) cytosines to uracil using EZ DNA Methylation Lightning Kit (Zymo Research). Samples were PCR amplified in four individual 10‐μL reactions with KAPA HiFi HotStart Uracil+ ReadyMix (Kapa Biosystems) and universal Illumina PE PCR primers. The PCR products were combined and purified together using a PCR cleanup kit and solid‐phase reversible immobilization beads. Library quality and quantity (>2 nM) was checked using a PicoGreen dsDNA Assay Kit and qPCR, while the consistency of the size profiles (150–600 bp with a peak around 450) was determined using a Bioanalyzer (Agilent; 5067‐4626). Pooled libraries were sequenced at Novogene (UK) Ltd. on the Illumina HiSeq4000 platform in 2‐ by 150‐bp paired‐end mode distributed across multiple sequencing lanes.

### 2.3 Reference sequence

The IBSCv2 barley genome assembly (Mascher et al., 2017) was used, which contains seven pseudochromosomes (chr1H to chr7H) plus an additional genomic sequence (chrUn) with concatenated unassigned scaffolds. As all of the seven barley pseudochromosomes exceed the 512 Mb size limit of bai indexing by SAMtools (Li et al., 2009), a custom Unix script was applied to split long chromosomes at the center, while avoiding splitting within annotated features. Split pseudochromosomes were used as references throughout all steps of the present experiment. Feature coordinates obtained on split pseudochromosomes were recalculated to original coordinates using another custom script.

### 2.4 Gene model

RNA sequencing reads representing 150 barley leaf samples (including the 12 barley lines of the present study) were mapped onto the split‐chromosome version of the IBSCv2 barley genome using HISAT2 (Kim, Langmead, & Salzberg, 2015). Transcript structures were reconstructed using StringTie (Pertea et al., 2015) and high‐quality exon junction data were obtained using the Portcullis tool (Mapleson, Swarbreck, Venturini, & Kaithakottil, 2018). These data, along with the published annotation data of the IBSCv2 assembly, were used to produce a polished gene model with Mikado pipeline (Venturini, Caim, Kaithakottil, Mapleson, & Swarbreck, 2018).

### 2.5 Identification of repetitive DNA sequences

Transposable elements and repeats were identified using RepeatMasker (version open‐4.0.6, http://www.repeatmasker.org) using the *Liliopsida* species model and RepBase Update 20160829. A custom pipeline was used to annotate the RepeatMasker output including the following main steps: (i) assign RM class and target to each RepeatMasker hit, (ii) merge overlapping features of identical class and target categories, and (iii) produce separate BED format files for each RM class category to enable intersecting methylation features and RM features.

### 2.6 Restriction fragment representation assessment

To assess the proportion of genomic regions covered by bisulfite‐treated short reads originating from PacI‐NsiI restriction fragments, sites of a minimum read depth of 5 were collected from bam‐format alignments after removing PCR‐duplicates using the coverage command in the BamTools toolkit (Barnett, Garrison, Quinlan, Strömberg, & Marth, 2011). Contiguous regions were created for intersecting and size estimation using the merge function from BEDTools (Quinlan & Hall, 2010).

### 2.7 Read alignment and measuring methylation level

Paired‐end reads were demultiplexed and barcode sequences were trimmed using the first script of the WellMeth pipeline WM‐1_preprocessing.sh. After demultiplexing and quality filtering, bisulfite‐treated short reads were aligned to the split‐chromosome version of the barley reference genome version Hv_IBSC_PGSB_v2 using WM‐2_mapping.sh. The second step also included removal of the PCR duplicates. Reads that were left after quality control and PCA duplicated methylation variants were called by the execution of the third script WM‐3_call_methylation.sh (Supplemental Figure S1). Methylation information ratios (number of methylated regions vs. all reads) for each cytosine were obtained in three sequence contexts (CG, CHG, and CHH). Following mapping and calling of the raw sequences of the methylation variants, data were filtered and only sites with minimum 5× read coverage were considered for further analysis.

### 2.8 Identification of differentially methylated sites and regions

Differentially methylated sites and regions were identified by all‐to‐all pairwise comparison of methylation patterns of the 12 barley cultivars using the WellMeth script WM‐4_diff_methylation.sh that implements a hidden Markov model–based differential methylation detection framework adopted from the BisulFighter package (Saito, Tsuji, & Mituyama, 2014).

To compare the level of methylation within DMRs at the population level, consensus regions were called using the merge function from BEDTools (Quinlan & Hall, 2010). Only regions with a minimum of five cytosines and a frequency (number of methylated cytosines per length of the DMR) higher than 0.2 were kept for further analysis. The level of DNA methylation was averaged independently for each region and cultivar.

### 2.9 Relatedness analysis

Total RNA was isolated from the same pool of seven flag‐leaf samples described above using the Sigma–Aldrich Total Plant RNA kit. The Beijing Genomics Institute performed RNA sequencing library construction and sequencing. Paired‐end sequencing was performed on the Illumina HiSeq4000 platform (2‐ by 100‐bp, ∼20 M reads per sample). Clean reads were mapped on the barley reference genome using HISAT2 (Kim et al., 2015). Alignments were subsequently processed by StringTie (Pertea et al., 2015) and normalized read count data (fragments per kilobase million [FPKM]) were collected for each sample for 80,855 transcripts. The FPKM values were then log2+1 transformed in order to stabilize the variance. The SNP variants were called from the bam alignments using a haplotype‐based variant detector Freebayes (Garrison & Marth, 2012). The SNPs were filtered using the R package SNPrelate (Zheng et al., 2012), where SNPs in high linkage disequilibrium (*r* ^2^ > 0.9) and >30% of missing values were filtered out, leaving a set of 72,642 polymorphic SNPs that were used to perform relatedness analysis.

### 2.10 Statistical analysis

A Mantel test was conducted with the VEGAN package (Dixon, 2003) in R to explore the correlation between epigenetic and genetic distance and between epigenetic distance and gene expression variation. For the Mantel test, Euclidean distance matrices were calculated for genetic markers, transformed FPKM values, and methylation data sets. The significance of the correlations from the Mantel tests was determined through the Pearson product–moment correlation coefficient after 9,999 permutations. Principal component analysis (PCA) was performed in R using the PCA function from the mixOmics package (Rohart, Gautier, Singh, & Cao, 2017).

### 2.11 Gene ontology term enrichment

To investigate the biological functions of genes with DMR within the gene body or promoter region, GO term enrichment analysis was carried out with the Python library GOATOOLS (Klopfenstein et al., 2018). Gene ontology–term categories that were statistically overrepresented in the differentially methylated regions compared with the total set of 13,433 genes covered by the sequencing were corrected for multiple comparisons with a false discovery rate of 0.05 using the Benjamini–Hochberg method (Benjamini & Hochberg, 1995).

## 3 RESULTS

### 3.1 Restriction enzymes

For double‐digestion, methylation insensitive, frequent (NsiI) and rare cutter (PacI) were selected based on expected coverage with 2 million reads. The summed length of genomic regions in the bwa‐meth alignments covered short reads with a minimum read depth of 5,and varied between 25 and 28 Mbp.

### 3.2 General characterization of the spring barley methylome

We performed global methylome analysis of the 12 spring barley cultivars using RRBS. The design captured 1.4 million reads (0.7% of the barley genome) and 3.2 million unique methylated sites among which 20% (0.67 million) were cultivar specific. Interrogation of the conservation of methylated sites between cultivars showed that 0.38 million methylated sites were consistently methylated in 90% of the cultivars, and only these sites were considered for any statistics and summary data of the analyzed population (Supplemental Table S1). Out of all 19 million cytosines covered by the analysis, 27% were fully methylated. In the analyzed barley cultivars, the CHH context was identified as the predominant type of DNA methylation contributing to >70% of total methylated cytosine, while 14% were in the CG and CHG contexts. Globally, in all cultivars, we found DNA methylation levels of 88, 74, and 49% in the CG, CHG, and CHH sequence context, respectively (Supplemental Table S1).

### 3.3 Genome‐wide distribution of DNA methylation

Mapped fragments were relatively equally distributed along the chromosomes, and the proportion of methylated cytosines captured by the analysis reflected the genome architecture (Supplemental Figure S2). Methylation within the gene bodies occurred at the highest densities in the distal chromosome regions that are characterized by a high gene copy number. Methylation within retroelements dominated at a greater distance from genes toward the centromeric regions, which follows the occurrence of the LTR retrotransposons (Mascher et al., 2017). The higher density of cytosine methylation within transposons was found in distal chromosome regions, mirroring the proximity to genes (Supplemental Figure S2).

Analysis of the genomic features of the methylated sites showed that 72% of methylated cytosines were found in repetitive DNA of which 65% were retroelements, particularly in LTR *Gypsy* (Supplemental Figure S3; Supplemental Table S2). About 3.5% of the methylated cytosines were found in gene bodies (including introns, exons, and untranslated regions [UTRs]) that directly reflect the proportion of the genic space in the barley genome (Wicker et al., 2017). Over 13% of methylated sites were located in the flanking regions specified 5 kb up‐ and 2 kb downstream of the genes and so‐called intergenic regions (between 5–100 kb upstream of genes). Approximately 11% of methylated cytosines could not be classified to any genomic feature, which is unsurprising considering 16% of the barley genome remains unannotated (Wicker et al., 2017).

**FIGURE 1:**
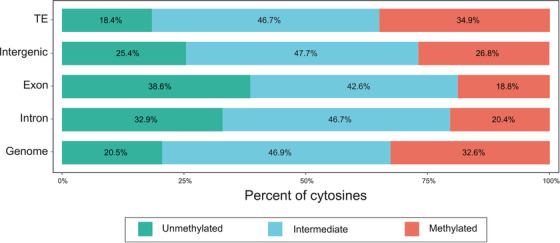
The percentage of methylated cytosines in the three main genomic regions: transposable elements (TEs), intergenic, genic (intron, exon), and the overall covered region. Each locus that could be identified in at least six cultivars was classified into three methylation levels: unmethylated (<10%), intermediate (>10% but <90%), and methylated (>90%)

Genic regions (introns and exons) showed a substantially high proportion of unmethylated cytosines (33–38%; Figure 1) and a low fraction of highly methylated sites. In comparison, transposable elements showed almost reversed proportions with 18% of cytosines (coverage > 4) having zero or very low methylation (down to 18%) and over 34% of cytosines being highly methylated (Figure 1).

**FIGURE 2:**
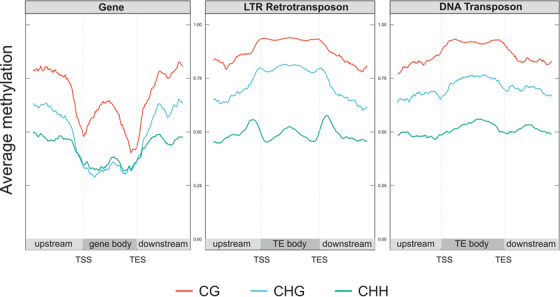
Methylation level distribution of CG, CHG, and CHH context along genes, retroelements, and DNA transposons. Upstream and downstream regions are proportionally the same length as the features

Methylation profiles were examined in all three sequence contexts within the gene body (i.e. introns, exons) and its flanking regions (up to 100% of the gene length) as well as within and around transposons and retrotransposons (up to 100% of the transposable elements [TEs] length) focusing on the three superfamilies: DNA *Cacta*, LTR *Gypsy*, and LTR *Copia*. The average methylation level (Table 1) and distribution (Figure 2) showed overall reduced DNA methylation in gene bodies and extensive methylation of transposable elements. For each feature, we identified three different methylation patterns. Genes were characterized by moderate CG and very low CHG and CHH methylation within the gene itself and increased level in the surrounding area (Figure 2a). The methylation level in all three contexts decreased sharply with proximity to genes, reaching a minimum at transcription start sites (TSS) and transcription end sites (TES). Many species show a similar trend of low (or no) gene body methylation in the CHG and CHH context (Niederhuth et al., 2016) but in the majority, the occurrence of CHH is significantly lower than CHG. Our results showed that generally, genes have a very similar level and pattern in both CHG and CHH context (Table 1, Figure 2). A very high level of methylation and its increase in comparison to the flanking regions in the CG context characterized both classes of transposable elements (Table 1, Figure 2). Differences in the methylation patterns between retrotransposons and DNA transposons were visible in the CHG and CHH context. The LTR retrotransposons were characterized by moderate peaks of CHG methylation and robust peaks of CHH methylation at the edges of TE (Figure 2b), while the pattern in DNA *Copia* showed only a moderate to low increase within the transposon body and no peaks in the flanking region (Figure 2c).

**TABLE 1:** The average methylation level according to local annotation and sequence context. Upstream or downstream flanking regions are defined as 5 kbp before transcription start sites or 2 kbp after transcription end sites

Features	Mean methylation level		
	CG	CHG	CHH
Flank (downstream)	0.70	0.51	0.42
Flank (upstream)	0.77	0.61	0.47
Gene body	0.60	0.35	0.36
Intergenic	0.90	0.75	0.47
Retroelement	0.94	0.80	0.46
DNA transposon	0.93	0.75	0.52
Unclassified	0.87	0.70	0.47

### 3.4 Methylation variation between cultivars

**FIGURE 3:**
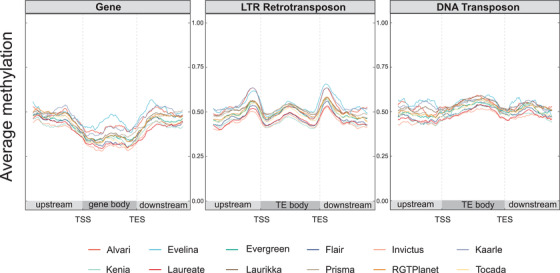
Methylation level distribution in CHH context along genes, retroelements, and DNA transposons. Upstream and downstream regions are proportionally the same length as the features

Methylation patterns in analyzed cultivars were not significantly different when the proportion of methylated sites in each context was considered (Supplemental Table S3). Average methylation of each cultivar was also comparable (Supplemental Table S1); however, differences could be observed in methylation of different genomic features (Supplemental Table S3). Cultivar‐specific differences in methylation patterns were particularly evident in the gene body and LTR retrotransposons (Supplemental Figure S4; Figure 3). In the genic and its flanking regions, three cultivars (Alvari, Kaarle, and Evelina) were separated from the rest and showed increased methylation especially in CG context (Supplemental Figure S4). There was an interesting dynamic between cultivars in CHH methylation in the immediate flanking regions of LTR retrotransposons; its methylation pattern separated cultivars into three groups (Figure 3). In the first group, Alvari, Evelina, and Kaarle showed the most striking peaks of CHH methylation at the edges of the LTR. Cultivars in the second group, Invictus and Laureate, had the lowest methylation levels in both retrotransposon body and surrounding area. The rest of the cultivars showed intermediate levels between the two groups.

### 3.5 Genetic diversity of the twelve cultivars

**FIGURE 4:**
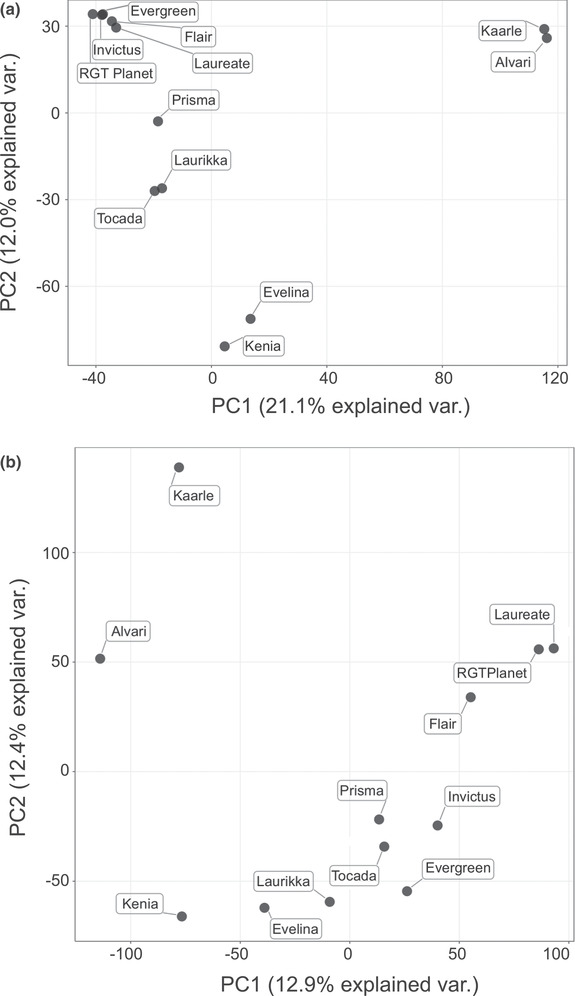
(a) Principal component analysis (PCA) showing the first and second principal components based on 72 642 SNPs identified from transcriptomes; (b) PCA showing the cultivars clustering based on normalized (log2+1 transformed) transcript expression data for the 12 cultivars and a total of 80855 transcripts

Genetic relatedness was analyzed using a subset of 72,642 polymorphic SNPs identified in transcribed regions. The first three principal components (PCs) explained 42% of the variance. Based on these components, the cultivars clustered into three groups (Figure 4a). The most distant cultivars were Kaarle and Alvari. Kenia and Evelina were gathered together and separated from the Finnish lines on both PCs. The additional group consisted of high‐malting cultivars for Scandinavian countries and northern Europe. The second PCA (Figure 4b) was performed using the transformed FPKM values for the transcript expression data. The first three PCs explained 34.5% of the variance (Figure 4b), which is less than for PCAs based on SNP data. The cultivars separated into three clusters, with Kenia and Evelina in one group, the Finnish cultivars Alvari and Kaarle in another, and the remaining (conventional and more recently released) cultivars in a third group.

### 3.6 Differential methylation

**FIGURE 5:**
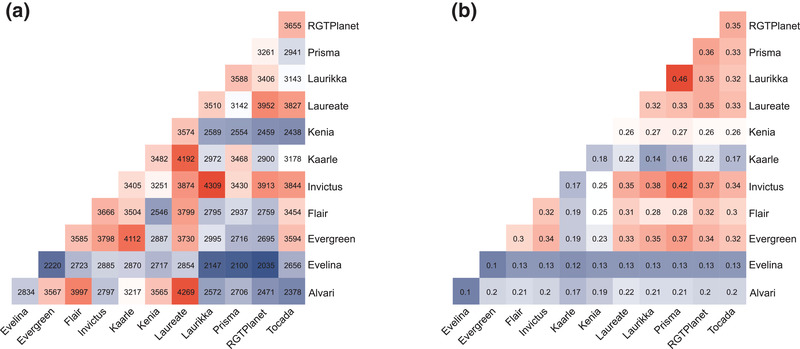
Methylation differences between different cultivars. Numbers of (a) differentially methylated regions and (b) differentially methylated sites (in millions) identified between each pair of cultivars

Differential methylation analysis between each pair of cultivars was conducted to compare methylation patterns of the commercial cultivars. We identified nearly 21,000 unique DMRs and 0.7 million DMCs between all the pairs of cultivars analyzed. On average, over 3,172 significant DMRs and 0.3 million DMCs could be detected between each pair. On the extremes, over 4,300 DMRs were identified between Laurikka and Invictus, while only 2,035 DMRs were identified between Evelina and RGTPlanet (Figure 5a). There were over 0.46 million DMCs identified between Prisma and Laurikka, while only 0.1 million DMCs were identified between Evelina and Evergreen or Alvari. Many DMRs did not necessarily match a high number of DMCs for each pair (Figure 5b). The limited overlap between DMCs and DMRs can explain this discrepancy. Only 45% of DMCs were found within regions that showed significant differences, the remaining DMCs (independent to DMRs) were scattered across the covered proportion of the genome. The majority of DMRs (78%) and DMCs (53%) were in CHG context. This is in contrast to the overall proportion of methylated sites, where CHH methylation was the most abundant with CG and CHG comprising <30% of all identified methylated sites. Only ∼1% of DMRs and 4% of DMCs identified between all pairs of cultivars were in CHH context.

### 3.7 Epigenetic diversity of the twelve cultivars

**FIGURE 6:**
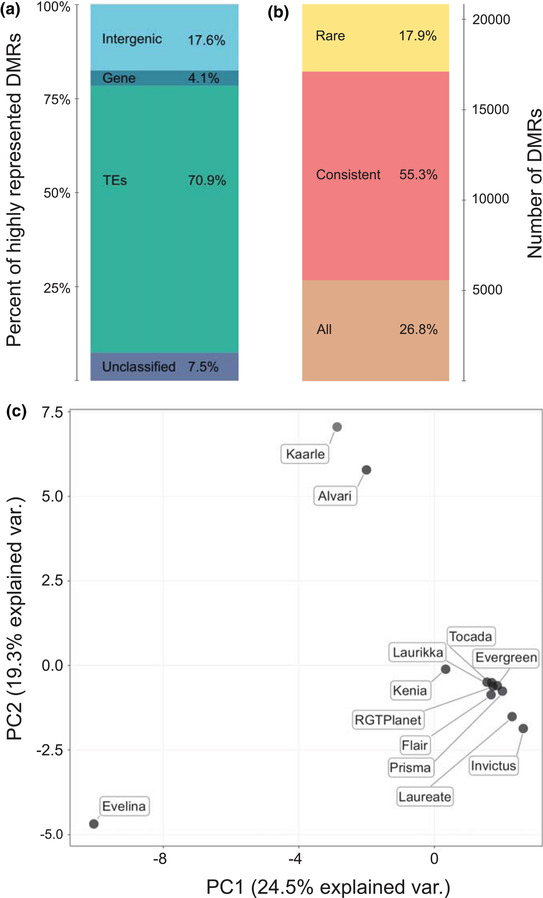
Methylation differences between different cultivars. (a) The number of differentially methylated regions (DMRs) identified in pairwise comparisons between each cultivar in the analyzed population. The DMRs were considered rare when <50% and rare when >50% of cultivars were methylated in the region. (b) Genome‐wide distribution of highly represented DMRs (regions methylated in all cultivars). (c) Summary of a principal component analysis showing the first and second principal components based on the average methylation in 5,578 DMRs that were consistently methylated in all cultivars

We evaluated the consistency of methylation within regions to investigate whether the number of DMRs between cultivars was due to real differences or similarities or variation in coverage. Differentially methylated sites methylated in at least six cultivars (≥50%) were classified as consistent, those occurring in less than six cultivars (<50%) as rare (Figure 6a), and regions methylated in all cultivars were classified as highly represented. The majority (82.1%) of 20,828 DMRs were consistently methylated in at least 50% of the cultivars, out of which 5578 were methylated in all of them (Figure 6a). Nearly 18% of the highly represented DMRs overlapped the intergenic regions and over 4% occurred within the gene bodies. Both proportions were significantly higher when compared with the distribution of the methylated loci in the genome (Figure 6b; Supplemental Figure S2). The PCA was performed using the average methylation in DMRs common for all cultivars (Figure 6c) to assess if methylation profiles could distinguish the cultivars and mirror their genetic diversity. The three first components described over 57% of the variance. In the middle of PC2 was a cluster of nine cultivars including Kenia that was coupled with Evelina in the genetic variance analysis.

Similar to the PCA of the transcriptome variants, Kaarle, Alvari and, Evelina were divergent from the main group. On the other hand, Kenia differed more from the main cluster based on the genetic variants than on the differential methylation pattern. The similarities and differences between genetic variants and methylation of highly represented regions were echoed in the transcript expression data. A Mantel test with 9,999 permutations showed that DNA methylation differences between the lines are significantly associated with DNA sequence differences (*R* = .62, *P* = .02). The test also revealed a positive correlation between epigenetic distance and gene expression variation (*R* = .54, *P* = .02). Expression differentiation of the analyzed cultivars was also significantly correlated with its genetic distance (*R* = .73, *P* < .001).

### 3.8 Gene ontology of methylated genes in barley cultivars

The genes that were covered by the sequenced reads in the promoters (1 kbp upstream of the TSS) and gene body regions were taken into consideration and termed as the methylated genes. The analysis covered over 13,000 genes. A total of 2,118 methylated genes (of the 13,433 genes covered in total by the RRBS) overlapped with DMRs within the gene body and promoter region, of which 467 were identified in all 12 cultivars. Gene ontology term enrichment analysis of functional domains in these 467 genes identified 17 significantly enriched GO terms, including GO terms associated with chromatin structure and organization and metal ion transport (Supplemental Figure S5).

## 4 DISCUSSION

In this study, we assessed the DNA methylation landscape at single‐base resolution in 12 commercial and historical spring barley cultivars developed for the European climate. Our results revealed features regarding the distribution of methylated cytosines, differential methylation patterns, and their relationship with genetic diversity. Furthermore, we offer a reproducible and complete pipeline to analyze epiGBS data for species with a high‐quality genome

The RRBS approach is a cost‐effective alternative to WGBS for large experimental designs and species with a large genome such as barley. We selected a combination of methylation insensitive, rare, and frequent cutting restriction enzymes, thereby avoiding bias toward any particular genic regions. Identified fragments were distributed equally and randomly along the chromosomes, which allowed for the representative identification of potential DMRs of interest found mostly outside of genes.

### 4.1 DNA methylation patterns in spring barley

We found that spring barley cultivars analyzed had much higher levels of methylation than other members of the Poaceae family, like rice (*Oryza sativa* L.) and maize (*Zea mays* L.), or in *Arabidopsis* (Cokus et al., 2008; Niederhuth et al., 2016; Figure 1 and 2; Table 1). Additionally, CHG and CHH methylation, which tends to be relatively low in most species, was unusually high in barley, with the level of methylation in CHH context being over 10‐fold higher than maize or green foxtail [*Setaria viridis* (L.) P. Beauv.] (Niederhuth et al., 2016). We also observed that the majority of cytosines methylated throughout the barley genome were in CHH context, similar to sugar beet (*Beta vulgaris* L. subsp. *vulgaris*) (Zakrzewski et al., 2014). These two trends are likely a consequence of the barley genome size, frequency of each context (Wicker et al., 2017), TEs composition, and mechanisms by which CHG and CHH methylation are established and maintained. Variation in methylation patterns between species has been observed before (Niederhuth et al., 2016; Takuno, Ran, & Gaut, 2016). Non‐CG methylation shows relatively little conservation at the level of individual methylation pathways, which seem to be lineage specific (Li et al., 2014; Takuno et al., 2016; Zemach et al., 2013). Despite the general similarities between plant species, uniquely high non‐CG methylation in barley suggests significant distinction in the predominance of methylation pathways and a species‐specific distribution of CHH in the genome.

### 4.2 DNA methylation patterns in genic regions

In contrast to heavy methylated transposable elements and intergenic regions, DNA methylation in gene bodies was decreased in all three contexts. Similarly to poplar (*Populus trichocarpa* Torr. & A. Gray), rice, or *Arabidopsis* (Feng et al., 2010; Zemach, McDaniel, Silva, & Zilberman, 2010), the CG methylation profile at the start and end of the gene body sequence colocalized with a prominent dip in average methylation. We found that the level of non‐CG methylation was low throughout the gene body, similar to observations in rice and soybean [*Glycine max* (L.) Merr.] (Garg, Narayana Chevala, Shankar, & Jain, 2015; Song et al., 2013). Interestingly, although methylation in UTRs was at a very low level in all three contexts (Supplemental Table S4), it was still significantly higher than methylation in UTRs typically observed in many plants species (Feng et al., 2010; Garg et al., 2015; Song et al., 2013).

Even though it was suggested that gene body methylation regulates splicing and gene expression, its function in plants remains mostly unclear (Bewick & Schmitz, 2017; Candaele et al., 2014; Zhou et al., 2016). In contrast to previous reports, which hypothesize that preferential methylation of the gene body occurs at exons (Gelfman, Cohen, Yearim, & Ast, 2013; Takuno & Gaut, 2013), we found that in spring barley, levels of DNA methylation are higher in genomic regions encoding introns than the exon sequences. However, these correlations hold true only for CG methylation and not for CHG or CHH methylation, highlighting an invariably low level of methylation across introns, exons, and UTRs. The pattern and proportion of intron–exon methylation suggest that in barley there is selection against intragenic TE methylation, possibly to avoid its negative effect on gene expression. Alternatively, TEs present in introns could be targeted by CG and not non‐CG methylation. It may be that the unique CG methylation pattern in introns and exons is due to gene body methylation in spring barley prioritizing CG context and, similarly to rice, this may play an essential role in pre‐messengerRNA splicing (Wang et al., 2016).

### 4.3 Methylation of the transposable elements

Epigenetic silencing driven by DNA methylation and histone modification is central for the repression of transposition and expression of TEs (Law & Jacobsen, 2010). Transposable elements’ repressive activity of DNA methylation is particularly important in plants with large genomes, for example, maize or barley, in which the sheer abundance of transposable elements determine chromosome structure and genome organization (Anderson et al., 2019; SanMiguel et al., 1996; Wicker et al., 2017). In the analyzed cultivars, pericentromeric heterochromatin, as well as repeats and TEs containing euchromatin regions, were heavily methylated in all cytosine contexts (Supplemental Figure S2; Table 1; Mascher et al., 2017). What is more, the CHH methylation in both LTRs and DNA transposons reached up to 10‐fold the level of the average methylation in many plant species (Cokus et al., 2008; Song et al., 2013; Niederhuth et al., 2016; Noshay et al., 2019). We observed distinct non‐CG methylation profiles within and at the edges of retrotransposons and transposon families. The difference is likely a consequence of the abundance of the identified TE families, their chromosomal position, and chromatin configuration (Haag & Pikaard, 2011; Law & Jacobsen, 2010; Zemach et al., 2013). While <6% of the methylated positions that we identified were within DNA transposons (Supplemental Table S2) and located predominantly in the gene‐rich regions (Supplemental Figure S2), TE methylation occurred mostly within the retrotransposons LTR *Gypsy* and LTR *Copia*. These two families of retroelements comprise almost 50% of the barley genome (Mascher et al., 2017; Wicker et al., 2017). It has been shown that RNA‐directed non‐CG methylation pathways target the edges of LTR retrotransposons, often located close to expressed genes (Li et al., 2015, Zemach et al., 2013). However, in spring barley, nearly 60% of highly methylated sites were identified in LTR *Gypsy*, which is enriched in pericentromeric regions (Wicker et al., 2017). This suggests that while very high TE body methylation confirms preferential suppression of the transposable elements, the dynamics of non‐CG methylation at the edges of TEs must have a distinct role. A dramatic increase in CHG and CHH methylation at the boundaries of TEs has been discussed before (Li et al., 2015; Noshay et al., 2019; Wang, Liang, & Tang, 2018; Zemach et al., 2013). However, barley LTRs (borders are targeted by CHH methylation) are mostly located in heterochromatin and do not show lowered CG and CHG methylation (quite the opposite). We hypothesize that the peaks near LTR act as markers for long retrotransposons and partition between hetero‐ and euchromatin, rather than prevent the spread of these families.

### 4.4 Epigenetic and genetic diversity in an analyzed spring barley population

The genome shock hypothesis proposes that under stress, TEs can threaten the stability of the genome through relocation and insertion of new copies but this TE reactivation may also facilitate genetic adaptation (McClintock, 1984). Therefore, changes in TE methylation are particularly interesting in species with large genomes that harbor a high proportion of TEs such as barley. In the cultivars examined in this study, the number of DMRs that overlapped with transposons (Figure 6) indicates a possible degree of reversibility of TE methylation in this species. A balance between hypo‐ and hyper–TE methylation through the promotion of genome plasticity (Fedoroff, 2012; Raffaele & Kamoun, 2012) may be a driver of epigenetic and phenotypic diversity and potentially the ability to adapt to different environments. Alternatively, the differences between cultivars observed in the methylation profiles in transposons and retrotransposons, as well as DMRs and DMCs identified within TEs, might be simply a consequence of transposon‐based genetic diversity within the analyzed population (Singh, Nandha, & Singh, 2017).

Because of its genome size, the analysis of barley methylome at the single cytosines level can be expensive, cumbersome, and time‐consuming. Nevertheless, we have shown that epigenetic analysis using the data obtained from RRBS allows for the characterization of DNA methylation profiles and to capture differences between individuals. The 12 barley epigenomes compared in this study represent cultivars adapted to mostly Nordic climate and represent only a small subset of barley diversity. However, a high degree of TE polymorphism has been reported before between just four maize inbred lines (Anderson et al., 2019). Therefore, with over 80% of the genome consisting of transposable elements (Mascher et al., 2017) and numerous quantitative trait loci already found in TEs of a population of 233 spring barley genotypes (Abdel‐Ghani et al., 2019), it can be expected that sufficient TE polymorphism is present in the analyzed cultivars. Our findings emphasize the potential importance of the methylation of retrotransposons in the promotion of genome plasticity and epigenetic diversity.

Although <1% of the genome was captured in this study, we managed to identify over 20,000 unique DMRs across all cultivars. Very often, an identified DMR was unique for just one pair of cultivars. However, analysis of the methylation patterns within all identified regions indicated that significant fractions of sites were conserved between the cultivars, showing the degree of epigenetic variation within a population grown under the same conditions. A subset of these conserved regions showed fluctuating levels of methylation and overlapped with nearly 500 genes involved in various biological processes and molecular functions. We found no evidence that the differences in DNA methylation within those genes were associated with the changes in their expression. However, those genes exhibited enrichment in the categories associated with chromatin and cellular structure and organization and in the GO analysis (Supplemental Figure S5), suggesting the interdependence of gene expression and epigenetic marks. Nonetheless, in the present study, it is not possible to decipher whether differences in methylation are a cause or a result of gene expression.

The interplay between genetic and epigenetic variation is yet to be fully understood. Various studies have demonstrated that the relationship is not straightforward and that multiple factors play a role in DNA methylation variation including genetic variants (Dubin et al., 2015; Richards, 2006; Richards, Bossdorf, & Verhoeven, 2010). In this study, PCA (Figure 4, 5 & 6) and Mantel tests revealed a significant relationship between epigenetic and genetic variation. To some extent, methylation profiles mirrored the genetic diversity of the cultivars, which was particularly visible in the separation of Finnish from Nordic lines and central European cultivars. Genetic, transcriptomic, and epigenetic distance similarities show that in the absence of the pressure of differential growth conditions, environmental cues cannot mask the effect of genetic relatedness and variability on methylation patterns. The number of DMRs conserved between the cultivars together with a relatively strong relationship between genetic and epigenetic distance suggests that a proportion of methylated regions are genetically controlled and should, therefore, be stably inherited across generations. This hypothesis can be supported by the fact that even though CHH was the most abundant methylation context, the majority of DMR and DMCs were in CG and CHG context. It has been demonstrated before that CG and CHG methylation is more stable in plant genome than CHH context in both responses to stress and daily methylation fluctuations (An et al., 2017; Liang et al., 2019). Therefore, we can believe that methylation differences between cultivars found mainly in CG and CHG context are, in fact, a consequence of genetic diversity within the analyzed population.

Once disentangled from genetic variation, the barley methylome may be a remarkable resource to identify epigenetic associations with phenotype, environment, and gene expression, acting as a powerful future tool to aid plant breeding. More extensive studies with more cultivars, various environments and several generations will be needed to find heritable methylation markers that could act as predictors of plant performance.

### 4.5 WellMeth, a complete, reduced representation bisulfite sequencing analysis pipeline

In this paper, we present WellMeth, a new RRBS analysis pipeline. The design of adapters and primers and the library construction principles of WellMeth are adopted from the epiGBS pipeline (Van Gurp et al., 2016), applying 4‐ to 6‐nt long asymmetrical barcodes on the forward and reverse adapters and a fixed number of random nucleotides (unique molecular identifiers [UMIs]) adjacent to the barcodes. The double‐stranded adapters used in WellMeth contain 5‐methylcytosine, except for an optional unmethylated C nucleotide that can be used for strand identification and assessment of bisulfite treatment efficiency. A combination of 12 forward and eight reverse adapters accomplish a 96‐plex design, thus reducing adapter‐related costs. The asymmetric barcode and UMI arrangement ensures equal sequence representation at the 5′ end of the sequenced fragments and minimizes phasing errors. While the epiGBS technology was originally designed as a reference‐free application, WellMeth is mainly recommended for large and complex genomes, for which high‐quality, annotated reference sequences (long scaffolds or pseudochromosomes) are available. Paired‐end sequencing technology and the combination of two methylation insensitive restriction enzymes is recommended. This allows for optimization of fragment size distribution and equal representation of different chromosome regions but does not create additional presence or absence differences resulting from methylation in restriction sites. As the forward and reverse adapters of each read pair carry a fixed combination of two different barcodes and two different cohesive ends, demultiplexing of sequence pools will yield double‐digested fragments only that will map in a prefixed orientation on genomic reference sequences. The UMI information can be efficiently used for postalignment identification of PCR duplicates.

WellMeth is based on the direct mapping of bisulfite‐treated short reads to genomic reference sequences. Its downstream analysis steps are entirely different to those of the epiGBS pipeline: WellMeth applies Je suite (Girardot, Scholtalbers, Sauer, Su, & Furlong, 2016) for fastq deconvolution, as well as for identification of PCR duplicates; bwa‐meth (https://github.com/brentp/bwa-meth) for mapping of bisulfite‐treated reads; and MethylDackel (https://github.com/dpryan79/MethylDackel) for calculating per‐base methylation metrics. The detection of DMCs and DMRs is carried out through pair‐wise comparisons using a hidden Markov model–based framework provided by the ComMet tool of the BisulFighter package (Saito et al., 2014). The whole analysis procedure of WellMeth is organized into four overlapping modules: preprocessing, mapping, methylation variant detection, and differential methylation). Custom scripts, allowing step‐by‐step or fully automated execution, interconnect the modules. WellMeth is fast; bisulfite‐treated short‐read data representing up to 300 samples (or a full dataset of an Illumina sequencing run) can be mapped and analyzed within 3 d on a high‐performance Linux cluster. While being designed for reduced representation bisulfite sequencing analysis, upon modifications in the scripts of the preprocessing module (and keeping consistency with the downstream analysis modules) WellMeth can also be applied for whole‐genome bisulfite analysis. We have made the WellMeth manual, executables, and test data available (https://sourceforge.net/projects/wellmeth/).

## AUTHOR CONTRIBUTIONS

The study was conceived, designed, and coordinated by TA, KTK, CSJ, and LBE. Plant material was provided by SFS, KTK, BE, LK, AJ, and JDJ. CW and MM performed lab work. IN provided bioinformatics support and developed WellMeth pipeline. MM performed the analysis with contributions from AKR. The manuscript was written by MM and IN with contributions from AKR, CW, and SFS. All authors read and approved the final manuscript.

## CONFLICT OF INTEREST DISCLOSURE

The authors declare no conflict of interest. The founding sponsors had no role in the design of the study, in the collection, analyses, or interpretation of data, and the writing of the manuscript.

## Supporting information

Supplemental Figure S1. Summary of the WellMeth analysis pipeline
Supplemental Table S1. Comparison of the number of cytosines and methylation levels between cultivars for each context.
Supplemental Figure S2. The distribution of methylated cytosines within mobile elements and gene body plotted along each chromosome.
Supplemental Figure S3. Genome‐wide distribution of DNA methylation. The proportion of features with methylated cytosines.
Supplemental Table S2. Mobile elements methylation ratios, after Mascher et al. (2017)
Supplemental Table S3. Comparison of the methylation level between genic features for each cultivar
Supplemental Figure S4. Methylation level distribution in (A) CG, and (B) CHG contexts along genes, retroelements, DNA transposons for each cultivar.
Supplemental Figure S5. Barplot showing significantly enriched GO terms in the 467 genes with DMRs identified in all 12 cultivars.
Supplemental Table 4. Comparison of the number of cytosines and methylation levels between introns, exons and UTRs for each context.

## Data Availability

WellMeth detailed manual and executables, along with test data, are available on https://sourceforge.net/projects/wellmeth/. The raw data is openly available in European Nucleotide Archive at https://www.ebi.ac.uk/ena/data/view/PRJEB38305, reference number PRJEB38305
