# Supplementary material for: The cytosine methylation landscape of spring barley revealed by a new reduced representation bisulfite sequencing pipeline, WellMeth
Source: Plant Genome. 2020 Sep 14;13(3):e20049. doi: 10.1002/tpg2.20049 (PMC12807407; doi:10.1002/tpg2.20049)
Supplement: Supplementary file 1 [file TPG2-13-e20049-s001.docx]

SUPPLEMENTAL INFORMATION

**The cytosine methylation landscape of spring barley revealed by a new Reduced Representation BisulfiteSeq pipeline, WellMeth**

Marta Malinowska, Istvan Nagy, Cornelis A. M. Wagemaker, Anja K. Ruud, Simon F. Svane, Kristian Thorup-Kristensen, Christian S. Jensen, Birger Eriksen, Lene Krusell, Ahmed Jahoor, Jens Jensen, Lars Bonde Eriksen, Torben Asp.

Number of pages: 10

Number of Tables: 4

Number of Figures: 5

**Supplemental Figure S1.** Summary of the WellMeth analysis pipeline

**Supplemental Table S1.** Comparison of the number of cytosines and methylation levels between cultivars for each context. Total mC is the number of identified methylated cytosines within the features.

|  | Methylation level | | | | Number of methylated cytosines | | | |
| --- | --- | --- | --- | --- | --- | --- | --- | --- |
|  | average | CG | CHG | CHH | CG | CHG | CHH | total mC |
| Alvari | 0.54 | 0.87 | 0.70 | 0.45 | 190052 | 181243 | 1004433 | 1375728 |
| Evelina | 0.58 | 0.88 | 0.72 | 0.51 | 149800 | 143267 | 814679 | 1107746 |
| Evergreen | 0.55 | 0.86 | 0.70 | 0.47 | 370752 | 362274 | 1993271 | 2726297 |
| Flair | 0.53 | 0.86 | 0.70 | 0.44 | 371293 | 364054 | 1995663 | 2731010 |
| Invictus | 0.52 | 0.85 | 0.68 | 0.43 | 392096 | 387864 | 2123751 | 2903711 |
| Kenia | 0.55 | 0.87 | 0.69 | 0.45 | 181195 | 171049 | 957598 | 1674306 |
| Kaarle | 0.54 | 0.87 | 0.70 | 0.46 | 232303 | 220761 | 1221242 | 1309842 |
| Laureate | 0.52 | 0.86 | 0.68 | 0.43 | 360751 | 354457 | 1950951 | 2666159 |
| Laurikka | 0.57 | 0.86 | 0.71 | 0.49 | 348874 | 340690 | 1874913 | 2564477 |
| Prisma | 0.56 | 0.86 | 0.70 | 0.49 | 407334 | 399200 | 2187435 | 2993969 |
| RGTPlanet | 0.55 | 0.86 | 0.70 | 0.46 | 409441 | 404277 | 2217128 | 3030846 |
| Tocada | 0.54 | 0.86 | 0.69 | 0.45 | 305466 | 294382 | 1612291 | 2212139 |

**Supplemental Figure S2.** The distribution of methylated position within mobile elements and gene body plotted along each chromosome. The vertical line indicates approximate centromere positions, according to Mascher et al. (2017).

**Supplemental Figure S3.** Genome-wide distribution of DNA methylation. The proportion of features with methylated cytosines

**Supplemental Table S2.** Mobile elements methylation ratios, after Mascher et al. (2017)

|  |  |  |  | % of methylated features | % of TEs |
| --- | --- | --- | --- | --- | --- |
| **Mobile element** | | | | **71.5** | **100.0** |
|  | **Class I: Retroelement** | | | **65.2** | **91.2** |
|  |  | LTR Retrotransposon | |  |  |
|  |  |  | unclassified LTR | 0.02 | 0.03 |
|  |  |  | Cassandra | 0.00 | 0.01 |
|  |  |  | *Copia* | 5.17 | 7.23 |
|  |  |  | *Gypsy* | 59.4 | 83.1 |
|  |  | non-LTR Retrotransposon | |  |  |
|  |  |  | LINE | 0.58 | 0.82 |
|  |  |  | SINE | 0.02 | 0.02 |
|  | **Class II: DNA Transposons** | | | **5.80** | **8.12** |
|  |  | DNA Transposon Superfamily | |  |  |
|  |  |  | unclassified | 0.53 | 0.74 |
|  |  |  | CACTA superfamily | 4.59 | 6.43 |
|  |  |  | hAT superfamily | 0.05 | 0.08 |
|  |  |  | Mutator superfamily | 0.16 | 0.22 |
|  |  |  | PIF/Harbinger superfamily | 0.11 | 0.16 |
|  |  |  | Mariner superfamily | 0.31 | 0.43 |
|  |  | Helitron |  | 0.05 | 0.07 |
|  | Low_complexity | | | 0.01 | 0.01 |
|  | Satellite | | | 0.08 | 0.11 |
|  | Simple_repeat | | | 0.38 | 0.53 |
|  | Unknown | | | 0.00 | 0.01 |
|  |  |  |  |  |  |
|  | *Retro-TE/DNA-TE ratio* | | | *11.2* |  |

**Supplemental Table S3.** Comparison of the methylation level between cultivars for each genic features

|  | Flank (downstream) | Flank (upstream) | Gene Body | Intergenic | Retroelement | DNA Transposon | Unclassified |
| --- | --- | --- | --- | --- | --- | --- | --- |
| Alvari | 0.49 | 0.56 | 0.41 | 0.59 | 0.61 | 0.61 | 0.56 |
| Evergreen | 0.46 | 0.53 | 0.38 | 0.57 | 0.58 | 0.59 | 0.53 |
| Evelina | 0.51 | 0.57 | 0.42 | 0.60 | 0.60 | 0.60 | 0.58 |
| Invictus | 0.43 | 0.50 | 0.35 | 0.54 | 0.56 | 0.57 | 0.50 |
| Kenia | 0.47 | 0.52 | 0.37 | 0.56 | 0.58 | 0.59 | 0.52 |
| Laurikka | 0.48 | 0.54 | 0.39 | 0.57 | 0.59 | 0.61 | 0.54 |
| Prisma | 0.47 | 0.53 | 0.39 | 0.57 | 0.58 | 0.59 | 0.53 |
| Tocada | 0.47 | 0.53 | 0.38 | 0.57 | 0.58 | 0.59 | 0.53 |
| Flair | 0.47 | 0.53 | 0.38 | 0.56 | 0.58 | 0.59 | 0.52 |
| Kaarle | 0.50 | 0.57 | 0.40 | 0.60 | 0.61 | 0.62 | 0.57 |
| Laureate | 0.45 | 0.51 | 0.36 | 0.55 | 0.57 | 0.58 | 0.51 |
| RGTPlanet | 0.48 | 0.54 | 0.39 | 0.58 | 0.59 | 0.61 | 0.54 |

**Supplemental Figure S4.** Methylation level distribution in (A) CG, and (B) CHG contexts along genes, retroelements, DNA transposons for each cultivar. Upstream and downstream regions are proportionally the same length as the features.

**Supplemental Figure S5.** Barplot showing significantly enriched GO terms in the 467 genes with DMRs identified in all 12 cultivars. The x-axis shows the –log10(p) value and the y-axis describes the GO terms. The GO terms are characterised as a biological process (BP, red) or molecular function (MF, blue).

**Supplemental Table S4**. Comparison of the number of cytosines and methylation levels between introns, exons and UTRs for each context. Total mC is the number of identified methylated cytosines within the features.

| Context | Feature | Total mC | Mean Methylation |
| --- | --- | --- | --- |
| CG | Exon | 15684 | 0.49 |
|  | Intron | 25911 | 0.67 |
|  | UTR | 17101 | 0.42 |
| CHG | Exon | 17482 | 0.31 |
|  | Intron | 35545 | 0.38 |
|  | UTR | 18992 | 0.28 |
| CHH | Exon | 72522 | 0.31 |
|  | Intron | 180004 | 0.37 |
|  | UTR | 86467 | 0.33 |
